# The emerging role of MCPH1/BRIT1 in carcinogenesis

**DOI:** 10.3389/fonc.2023.1047588

**Published:** 2023-01-31

**Authors:** Mona Alsolami, Doaa Aboalola, Dolal Malibari, Tariq Alghamdi, Walaa Alshekhi, Hind Jad, Rea Rumbold-Hall, Ahlam S. Altowairqi, Sandra M. Bell, Rawiah Abdullah Alsiary

**Affiliations:** ^1^ King Abdullah International Medical Research Center (KAIMRC), King Saud Bin Abdulaziz University for Health Sciences, Ministry of National Guard - Health Affairs, Jeddah, Saudi Arabia; ^2^ Oncology Department, Princess Nourah Cancer Center, King Saud bin Abdulaziz University for Health Sciences, Ministry of National Guard - Health Affairs, Jeddah, Saudi Arabia; ^3^ Division of Molecular Medicine, Leeds Institute of Medical Research (LIMR), St James’s University Hospital, University of Leeds, Leeds, United Kingdom

**Keywords:** MCPH1, microcephalin, BRIT1, cancer, breast cancer

## Abstract

The MCPH1 gene, also known as BRCT-repeat inhibitor of hTERT expression (BRIT1), has three BRCA1 carboxyl-terminal domains which is an important regulator of DNA repair, cell cycle checkpoints and chromosome condensation. MCPH1/BRIT1 is also known as a tumour suppressor in different types of human cancer. The expression level of the MCPH1/BRIT1 gene is decreased at the DNA, RNA or protein level in a number of types of cancers including breast cancer, lung cancer, cervical cancer, prostate cancer and ovarian cancer compared to normal tissue. This review also showed that deregulation of MCPH1/BRIT1 is significantly associated with reduced overall survival in 57% (12/21) and relapsed free survival in 33% (7/21) of cancer types especially in oesophageal squamous cell carcinoma and renal clear cell carcinoma. A common finding of this study is that the loss of MCPH1/BRIT1 gene expression plays a key role in promoting genome instability and mutations supporting its function as a tumour suppressor gene.

## Introduction

### Overview

MCPH1 was identified as the first gene associated with primary microcephaly (MCPH). MCPH is an autosomal recessive genetic disorder that produces a smaller-than-normal head circumference at birth (1). MCPH1 has been referred to as BRIT1 (BRCT-repeat inhibitor of hTERT expression) due to its identification in a screen as a negative regulator of telomerase function (2). Mutations in MCPH1/BRIT1 were also found to cause premature chromosome condensation syndrome (PCC) in which cells demonstrate deregulation chromosome condensation (3).

### MCPH1/BRIT1 structure

**Figure 1:**
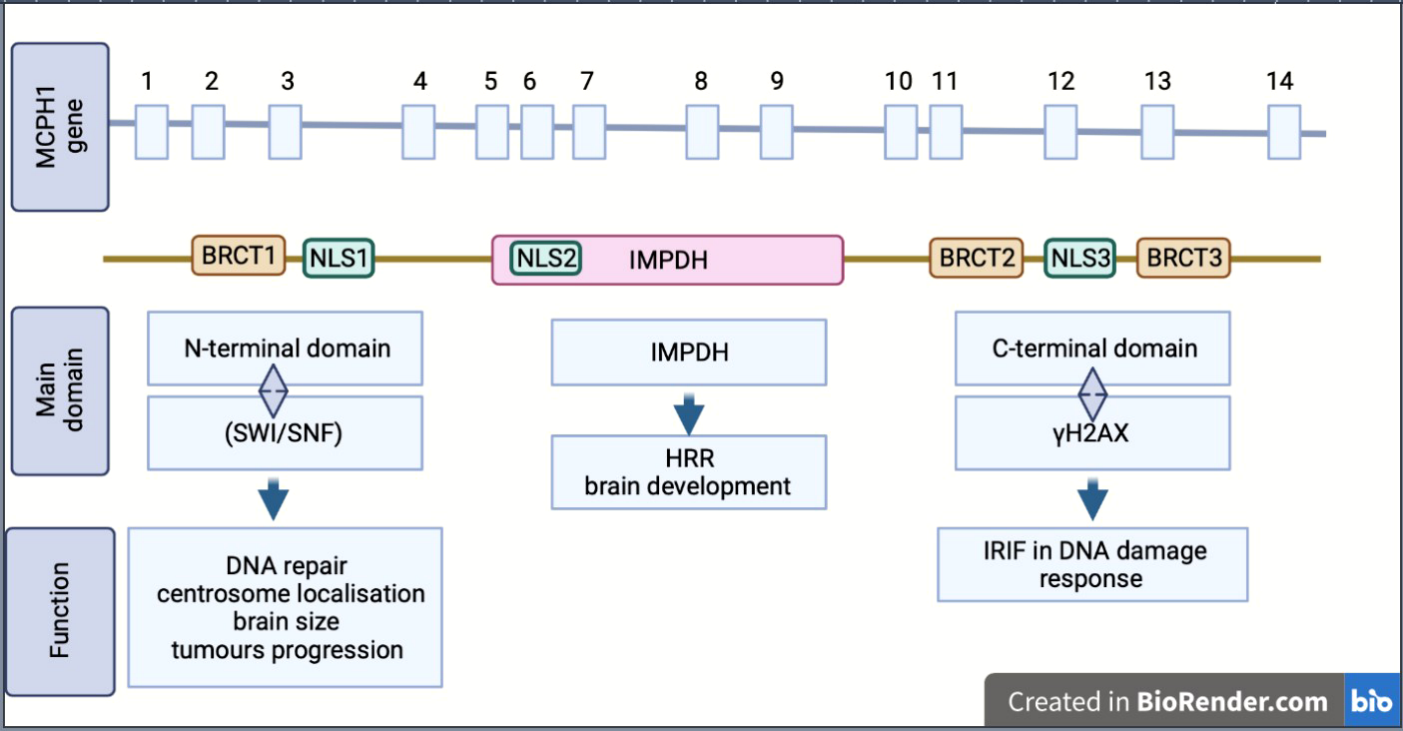
Schematic showing the structure, isoform and biological function of MCPH1/BRIT1. *MCPH1/BRIT 1* gene consist of 14 exon and express Microcephalin protein which comprises of three BRCT domains, separated by variable linker regions. BRCT domains play an important role in MCPH1/BRIT1 functions and protein-protein interactions. Inosine 5′-monophosphate dehydrogenase (IMPDH), Homologous recombination repair (HRR), Ionizing radiation induced foci (IRIF), nuclear localization signal or sequence (NLS), SWItch/Sucrose Non-Fermentable (SWI/SNF).

MCPH1/BRIT1 is located on chromosome 8p23; it contains 14 exons encoding an 835 amino acid (aa) protein called Microcephalin (**Figure 1**) (4). The MCPH1/BRIT1 protein comprises three BRCT domains with repeated 85–95aa long domains separated by variable linker regions no longer than 24aa (1). The first BRCT domain is located in the N-terminal of the MCPH1/BRIT1 protein between 2–83aa and the other two BRCT 2/3 domains lie between 320–399aa and 432-502aa, respectively in the C-terminal of the protein (5, 6). These domains play an important role in MCPH1/BRIT1 functions and protein-protein interactions (7) (**Figure 1**). Multiple sequence alignment of MCPH1/BRIT1 across H.sapiens, P.troglodytes, C.lupus, B.taurus, M.musculus, R.norvegicus, and G.gallus using ClustalX, revealed that the MCPH1/BRIT1 sequence is conserved (**Table 1**).

**Table 1:** Protein multiple alignment, pairwise similarity scores and evolutionary distances.

Protein accession	Organism	Sequence	Identity (%)	
NP_078872.2	H.sapiens	809 KYLSEKWVLDSITQHKVCAPENYLLSQ 835	**Protein**	**DNA**
NP_001009010.1	Vs. P.troglodytes	809 KYLSEKWVLDSITQHKVCASENYLLSQ 835	98.2	99.0
NP_001003366.1	Vs. C.lupus	824 WYLSETWILDSIIQHKVCAFDNYLLLH 850	63.8	75.0
NP_001096785.1	Vs. B.taurus	813 KYLSETWILDSISQHKVCASENHLLP- 838	58.6	67.9
NP_775281.2	Vs. M.musculus	796 QYLSEKWVLDSITQHKICDFNNYQLLQ 822	59.6	71.5
XP_006253445.1	Vs. R.norvegicus	782 QYLSEKWVLDSITQHKICDFNNYQLLQ 808	58.7	71.6
NP_001098789.1	Vs. G.gallus	487 KCLSEKWILDSITQHTVCPMENYIFQL 513	59.3	65.1

Multiple sequence alignment of MCPH1/BRIT1 across H.sapiens, P.troglodytes, C.lupus, B.taurus, M.musculus, R.norvegicus, and G.gallus using ClustalX, which revealed that the MCPH1/BRIT1 gene sequence is conserved. The last two columns displayed pairwise alignment scores of MCPH1/BRIT1 human vs all others. All data were adapted from National Library of Medicine.

Moreover, the association of these domains with protein function was reported in many studies; the N-terminal BRCT domain interacts with the chromatin remodelling complex (SWI/SNF) in DNA repair and associates with centrosome localisation (5, 7). This region is important in disease discovery due to its association with many mutations that arise in MCPH1/BRIT1; furthermore, many studies have suggested there is a link between N-terminal domains and determining brain size, gonad development and tumour progression (8, 9). The other domains, the tandem C-terminal BRCT domains, are essential for forming oligomer and ionizing radiation-induced foci (IRIF) by interacting with γH2AX in DNA damage response (5, 6). The central region of MCPH1/BRIT1, called the central microcephalin protein domain (IMPDH), is predicted to have a role in the homologous recombination repair (HRR) and brain development (6, 10).

### MCPH1/BRIT1 isoforms

The MCPH1/BRIT1 protein exists in multiple isoforms (variants), including the full length-MCPH1/BRIT1 that contains the three BRCT domains, the transcript MCPH1/BRIT1 Δe9–14, which is lacking the last six exons, the transcript MCPH1/BRIT1 Δe1–3, which lacks the first three exons and causes the drop of the N-terminal BRCT domain and finally the transcript MCPH1/BRIT1 Δe8 which is missing exon 8 resulting in the loss of the canonical nuclear localisation signal (NLS) motif (1, 2, 11) (**Figure 1**).

### MCPH1/BRIT1 expression

High expression of this MCPH1/BRIT1 protein was observed in the human foetal brain, testis, liver, pancreas and kidneys, while lower expression was reported in the heart, lungs, thymus and spleen (1). Many studies have reported that the expression level of the MCPH1/BRIT1 gene is decreased in different types of cancers including lung cancer, cervical cancer, breast cancer, prostate cancer and ovarian cancer when compared with normal tissues.

### MCPH1/BRIT1 function

**Figure 2:**
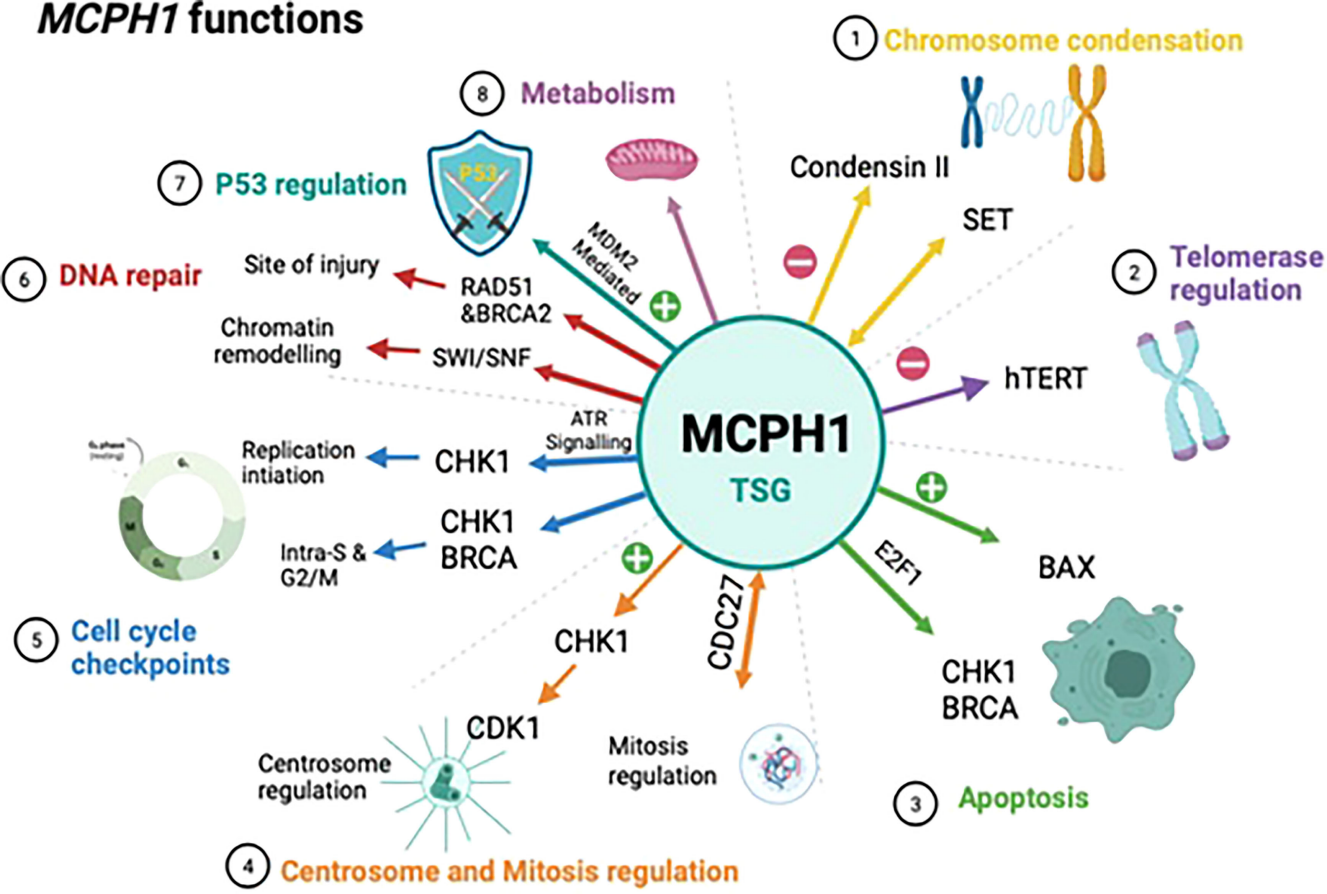
Schematic showing the biological functions of MCPH1/BRIT1. This schematic summarises the biological functions of MCPH1/BRIT1 which supports to its role as a tumour suppressor. 1) MCPH1/BRIT1 affects chromosomal condensation by inhibiting condensin II when it interacts with both non-SMC condensin II complex subunit D3 (NCAPD3) and non-SMC condensin II Complex Subunit G2 (NCAPG2) causing an abnormality in spindle structure and chromosome misalignment. 2) The telomere binding protein (TRF2) interacts with the MCPH1/BRIT1 protein forming the MCPH1/BRIT1–TRF2 complex which increases DNA damage factors localisation, resulting in repair of defective telomeres and increasing telomere replication. 3) MCPH1/BRIT1 promotes cell apoptosis and cell cycle arrest in the S and G2/M phases and overexpression of MCPH1/BRIT1 inhibits uncontrolled cell proliferation. 4) MCPH1/BRIT1 deficient cells demonstrate early mitotic entry which results in spindle organization abnormalities and chromosomal misalignment. 5) MCPH1/BRIT1 regulates the intra-S and G2/M cell cycle checkpoints. MCPH1/BRIT1 is required for adaptive response to avoid topoisomerase II inhibition-mediated G2 arrest and proper chromosomal alignment during prometaphase. 6) Mutations in MCPH1/BRIT1 could lead to defects in DNA repair and cancer development, since MCPH1/BRIT1 is required to recruit and maintain the BRCA2-Rad51 complex at the site of DNA injury and regulate homologous recombination repair of DNA DSBs in a BRCA2-dependent manner. 7) High MCPH1/BRIT1 expression stabilizes p53 function by inhibiting MDM2 and may impact chemotherapy response. 8) MCPH1/BRIT1 is crucial for controlling mitochondrial activity and participating in bioenergetic pathways including oxidative phosphorylation pathways.

MCPH1/BRIT1 performs a wide range of biological functions which supports its role as a tumour suppressor gene, these are outlined below and summarized in (**Figure 2**).

### Chromosome condensation

Neitzel et al. (2002) characterizes novel autosomal recessive condition with premature chromosomal condensation (PCC) in the early G2 phase in two microcephaly-affected consanguineous siblings with mental retardation. They noted a high frequency of prophase-like cells (>10%) in their lymphocytes, fibroblasts and lymphoblast cells. Neitzel postulated that this PCC is caused by early mitosis as a result of changes in these two siblings’ cell-cycle regulatory genes (12). Years later MCPH1/BRIT1 was discovered to be a chromosomal condensation regulator, inhibiting condensin II by interacting with both NCAPD3 and NCAPG2 subunits (11, 13). Previous research demonstrated that the MCPH1/BRIT1 mutation caused transcriptional alterations that significantly increased aberrant chromosomal condensation. When MCPH1/BRIT1 is not expressed, early entrance into mitosis was reported, which can result in abnormalities in the spindle’s structure and chromosome misalignment linked to premature chromosome condensation (PCC) (14–17).

### Telomerase regulation

Telomeres are repeating nucleotide sequences present at the ends of chromosomes that assist to protect the end of the chromosome from DNA damage (18, 19). Somatic cell telomeres shorten with each cell cycle (20). In cancer cells the activation of the telomerase enzyme inhibits DNA shortening in by adding repeated nucleotide sequences to the ends, which helps keep the cells alive (21). It has been reported that MCPH1/BRIT1 influences telomerase activity (2). Cicconi et al. revealed that the telomere binding protein TRF2 interacts with the MCPH1/BRIT1 protein. MCPH1–TRF2 complex increases localisation of DNA damage factors and directs repair of defective telomeres. MCPH1/BRIT1 also participates in the increasing telomere replication fork development and the initiation blocked telomere replication forks (19).

Telomerase enzyme consists of subunits, one of which is an RNA component (hTR) and the other a telomerase reverse transcriptase (hTERT) (22). MCPH1/BRIT1 was initially identified as a result of a genetic screen for transcriptional repressors of the human telomerase catalytic subunit (hTERT). Since hTERT and MCPH1 are negatively connected, MCPH1 is often referred to as BRIT1, which stands for (BRCT-repeats inhibitor of hTERT expression) (2). It might be as a result of direct interaction with the hTERT promotor, which would reduce hTERT expression and consequently lower telomerase activation (23).

Alternative splicing of the hTERT gene has been shown to impact the activity of the telomerase enzyme (hTERT) and contribute to a range of different diseases (24). Thyroid cancer, gastrointestinal carcinoma, ovarian cancer, and myelodysplastic disorders have all been linked to splice variants (25–28). According to a prior study, MCPH1/BRIT1 acts as a negative regulator of the functional variant α+/β+ hTERT in ovarian cancer, while, the α-/β+ hTERT and α-/β- hTERT variants have a positive relationship with MCPH1/BRIT1 supporting its function as a potent inhibitor of telomerase activity (27).

### Apoptosis

MCPH1/BRIT1 control apoptosis and mitosis entry by promoting cell apoptosis and arresting the cell cycle in the S and G2/M phases. Further investigation revealed that the CDC25C-cyclinB/CDC2, p53/p21, cyclinA2/CDK2, and CDC2 pathways were engaged in the S phase arrest induced by MCPH1/BRIT1 overexpression (29). Moreover, the overexpression of MCPH1/BRIT1 inhibits uncontrolled cell proliferation in combination with a significant increase in the levels of Bax and active caspase-3 and a decline in the level of Bcl-2 (30). Mai et al. reported that the overexpression of MCPH1/BRIT1 activated mitochondrial apoptosis through regulating several apoptosis-related proteins such as p53, cytochrome c and PARP-1 (29).

### Centrosome and mitosis regulation

Centrosomes in human cells are responsible for arranging microtubules, which enable a number of cellular functions such as cell polarization and the formation of the mitotic spindle to ensure proper chromosomal segregation during mitosis. Centrosome amplification or increase in centrosomes number, is frequent in cancer and is associated with more aggressive clinical characteristics. It was postulated that MCPH1/BRIT deficiency is one of the key reasons for centrosome amplification and increase in centriole number (16, 31). In presence of MCPH1/BRIT1, the N-terminal BRCT domain of MCPH1/BRIT1 is required for centrosomal localization in irradiated cells (6, 16). In MCPH1/BRIT1 deficient cells, early mitotic entry is seen, which results in spindle organization abnormalities and/or chromosomal misalignment (16).

### Cell cycle checkpoints

Cellular checkpoints regulate mitotic entrance by delaying the initiation of mitosis until the abnormality is completely repaired. MCPH1/BRIT1 activity is not necessary for maintenance of the decatenation checkpoint (32), however it has been recognized as a crucial cell cycle regulator (16). MCPH1/BRIT1 is a regulator of the intra-S and G2/M checkpoints. MCPH1/BRIT1 is necessary for the checkpoint kinase1 (Chk1) localization (17). In MCPH1/BRIT1 deficient cells, active Cdk1 is required for the premature onset of chromosomal condensation (33). MCPH1/BRIT1 is required for the adaptive response that avoids topoisomerase II inhibition-mediated G2 arrest (32). Furthermore, MCPH1/BRIT1 interacts with the anaphase-promoting complex *via* Cdc27, connecting transcription to cell cycle progression (16). MCPH1/BRIT1 is also necessary for proper chromosomal alignment during prometaphase (33).

### DNA repair

MCPH1/BRIT1 plays a role in DNA repair at the site of injury. DNA double strand breaks (DSBs) are harmful to cells and could lead to mutations and cancer progression if not repaired. The BRCA2-Rad51 complex repairs these DSBs and it has been shown that MCPH1/BRIT1 is required to recruit and maintain the BRCA2-Rad51 complex at the site of injury whilst DNA repair occurs (34). Furthermore, MCPH1/BRIT1 can regulate homologous recombination repair of DNA DSBs in a BRCA2-dependent manner. Therefore, reduction or mutation in MCPH1/BRIT1 could progress to defects in DNA repair and subsequently to cancer (34).

Chromatin remodelling is an area of DNA repair that involves MCPH1/BRIT1. DNA repair can only occur if DNA damage repair (DDR) proteins have access to detect and repair the damaged DNA, this cannot happen when the chromatin is condensed. ATP-dependent chromatin remodelling is used by cells to loosen chromatin and give access to DDR proteins (35). MCPH1/BRIT1 is a regulator of the ATP-dependent chromatin remodelling complex SWI/SNF. When DNA is damaged the interaction of MCPH1/BRIT1 with SWI/SNF is increased by the ATM/ATR-dependent phosphorylation of the BAF170 subunit. This increased interaction allows SWI/SNF to be recruited at the site of DNA damage and promotes chromatin relaxation whilst the recruitment of DDR proteins takes place (8). Loss of MCPH1/BRIT1 causes a decrease in chromatin relaxation through the reduced MCPH1-SWI/SNF interaction which could lead to cancer development (8).

### P53 regulation

In p53(-/-) cells, Mcph1/Brit1 deficiency resulted in significantly increased chromosomal and chromatid breakage, aberrant centrosome proliferation and aneuploidy (36). MCPH1/BRIT1 has been shown to increase p53 protein stability in breast cancer cell lines by inhibiting MDM2-mediated p53 ubiquitination (37). Hence, MCPH1/BRIT1 deficiency induces genomic instability and enhances cancer risk. The substantial pre-chemotherapy connection between MCPH1/BRIT1 and p53 verifies the concept that high MCPH1/BRIT1 expression stabilizes p53 function and may impact chemotherapy response (38).

### Metabolism

MCPH1/BRIT1 colocalize with GRP75 which interacts with the voltage-dependent anion channel 1 (VDAC1) in the mitochondria. VDAC1 protein is required for metabolite exchange between mitochondria and the intracellular environment, as well as calcium flow from the endoplasmic reticulum into mitochondria for tricarboxylic acid cycle activation (39–41). Hence, MCPH1/BRIT1 is crucial for controlling mitochondrial activity and participating in bioenergetic pathways including oxidative phosphorylation pathways (41).

## Method

### Inclusion and exclusion criteria for studies

We conducted a systematic scoping review to search and identify eligible studies in October 2022 by searching databases included MEDLINE, COCHRANE, EMBASE, Science Direct, and PUBMED. We used the “MCPH1/BRIT1 or Microcephalin” in combination with at least one of the following words: “cancer”, “tumour”, “tumor” and malignancy”. We included articles meeting the following criteria: the abstract was available, evaluating the potential role of MCPH1/BRIT1 in different types of cancers and articles published between years 2000 to 2022. We excluded non-English articles, and articles published before 2000.

### Data abstraction and reporting of results

The search for selected published articles was made by three authors (MA, SMB and RA). The summary of included studies is shown in **Table 2**. We performed an independent review of article titles and abstracts extracting data according to the predefined eligibility criteria. To assess the Quality of the selected studies we used the Newcastle–Ottawa scale (NOS) which scores a study with a total of 9 points according to the assessment criteria such as criteria for selection, comparability and outcome (case–control studies) or exposure (cohort studies) (70). A good quality study has 3 or 4 points in selection criteria, 1 or 2 points in comparability criteria, and 2 or 3 points in outcome/exposure criteria.

**Table 2:** Summary of studies on MCPH1/BRIT1 in human cancer.

Type of tumour	References
**Breast Cancer**	• Richardson et al. (42) • Y. H. Jo et al. (43) • Bhattacharya et al. (44) • Partipilo et al. (45) • Mantere et al. (46) • Tervasmäki et al. (47) • Claudia Cava et al. (48)
**Colorectal Cancer**	• Y. S. Jo et al. (49) • Xicola et al. (50) • Denu and Burkard (31) • Tricoli et al. (51) • *Du* Z et al. (52)
**Ovarian Cancer**	• Bruning-Richardson et al. (53) • Alsiary et al. (27) • Ria et al. (54) • Alsiary et al. (55)
**Lung Cancer**	• Zhang et al. (56) • Zhou et al. (30) • Wu et al. (57)
**Other type of cancer**	• Jo et al. (49) • Li et al. (58) • Hagemann et al. (59) • Venkatesh et al. (60) • Denu et al. (31) • Liang et al. (61) • Rai et al. (54) • Giallongo et al. (62) • Kopparapu et al. (63) • Mai et al. (29) • Bilbao et al. (64) • Liang et al. (65) • Wang et al. (66) • Ghodsi et al. (67) • Viet et al. (68) • Russo et al. (69)

## Results

### Perspective on the involvement of MCPH1/BRIT1 in cancer progression

**Figure 3:**
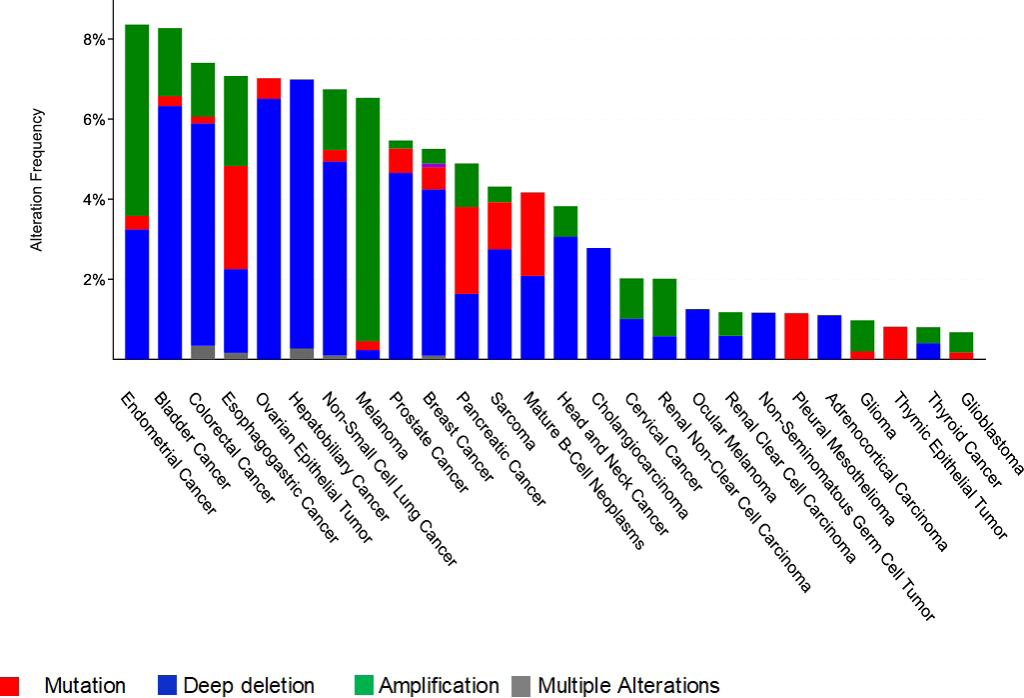
Frequency and type of genetic alterations at the *MCPH1/BRIT1* locus in different cancer types.

In this review we are interested in MCPH1/BRIT1 expression levels and its association with tumorigenesis in various forms of cancer, whether at the DNA, RNA or protein level. Interrogation of The Cancer Genome Atlas (TCGA; cBioPortal Cancer Genomics database http://cancergenome.nih.gov/) has identified alterations in MCPH1/BRIT1 in many malignant tumours such as breast cancer (~5%), prostate cancer (5.7%), bladder urothelial carcinoma (8%), lung cancer (7%), ovarian cancer (7%), liver cancer (7%) and colorectal adenocarcinoma (7.4%) (**Figure 3**). Deep deletions and mutations were the most common inactivation methods in most cancer types with amplifications in a minority of cancers, while structural variants were quite rare. This was confirmed in a study conducted on various types of tumours, which identified MCPH1/BRIT1 gene deletions in 5–15% of cancers (31).

**Figure 4:**
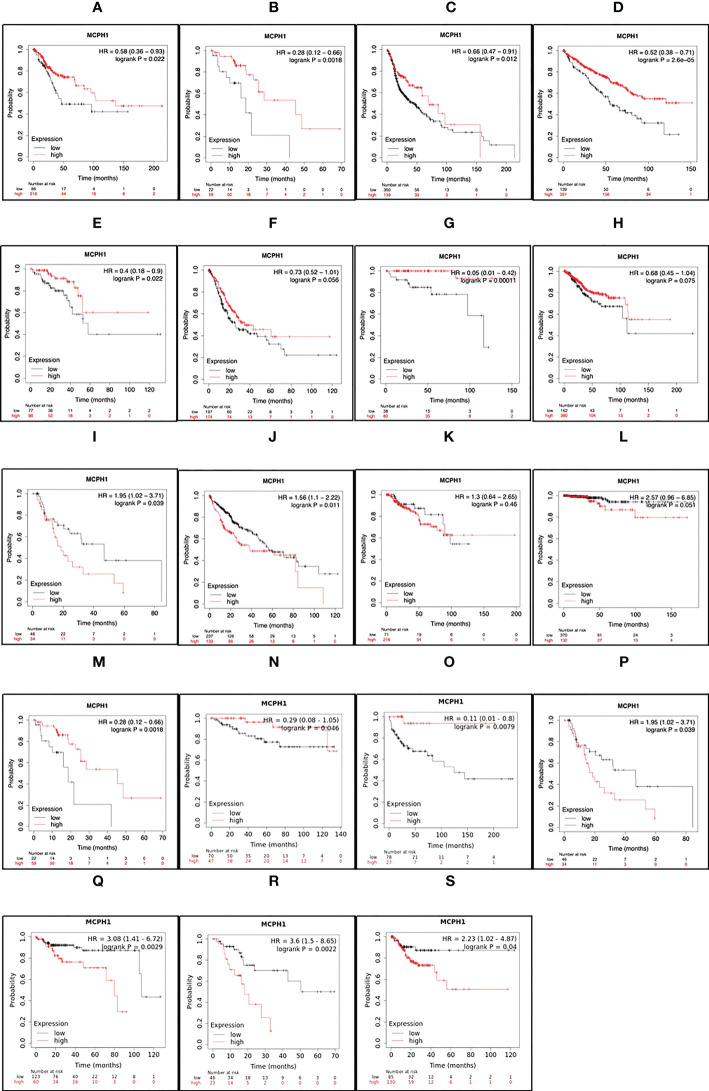
MCPH1/BRIT1 expression correlated with overall survival (OS) and relapse free survival (RFS). Low MCPH1/BRIT1 expression in cervical squamous cell carcinoma **(A)**, oesophageal squamous cell carcinoma **(B)**, head-neck squamous cell carcinoma **(C)**, renal clear cell carcinoma **(D)**, rectal adenocarcinoma **(E)**, stomach adenocarcinoma **(F)**, thymoma **(G)** and uterine corpus endometroid cancer **(H)** predicted reduced OS. High MCPH1/BRIT1 expression in oesophageal adenocarcinoma **(I)**, renal papillary cell carcinoma **(J)**, HCC **(K)**, and thyroid carcinoma **(L)** predicted reduced OS. Low MCPH1/BRIT1 expression in oesophageal squamous cell carcinoma **(M)**, renal clear cell carcinoma **(N)** and testicular germ cell tumours **(O)** predicted reduced RFS while high MCPH1/BRIT1 expressions in oesophageal adenocarcinoma **(P)**, renal papillary cell carcinoma **(Q)**, pancreatic ductal adenocarcinoma **(R)** and stomach adenocarcinoma **(S)** predicted reduced RFS.

To determine the clinical relevance of MCPH1/BRIT1 expression, pan-cancer analysis of RNA-SEQ data was performed using KM Plotter (71). MCPH1/BRIT1 expression was associated with overall survival (OS) in 57% (12/21) and relapse-free survival (RFS) in 33% (7/21) of cancer types (**Figure 4**). Low MCPH1/BRIT1 expression in cervical squamous cell carcinoma, oesophageal squamous cell carcinoma, head-neck squamous cell carcinoma, renal clear cell carcinoma, rectal adenocarcinoma, stomach adenocarcinoma, thymoma and uterine corpus endometroid cancer predicted reduced OS. Although high MCPH1/BRIT1 expression in a few cancers, oesophageal adenocarcinoma, renal papillary cell carcinoma, HCC, and thyroid carcinoma predicted reduced OS. Similarly, low MCPH1/BRIT1 expression in oesophageal squamous cell carcinoma, renal clear cell carcinoma and testicular germ cell tumours predicted reduced RFS while high MCPH1/BRIT1 expressions in oesophageal adenocarcinoma, renal papillary cell carcinoma, pancreatic ductal adenocarcinoma and stomach adenocarcinoma predicted reduced RFS (71).

## Discussion

### MCPH1/BRIT1 in breast cancer

Previously we have identified reduced MCPH1/BRIT1 expression in 29% (93/319) of breast cancer cases associated with high tumour grade and triple negative phenotype. Importantly MCPH1/BRIT1 was an independent predictor of overall survival (42). Furthermore, previous studies found MCPH1/BRIT1 to be associated in all subtypes (48). Another study identified low MCPH1/BRIT1 nuclear expression in 52.4% (43/82) of breast cancers which was associated with the presence of allele T in the MCPH1/BRIT1 polymorphisms (rs2912010 and rs1057090). This study also reported that increased MCPH1/BRIT1 cytoplasmic levels were associated with tumour grade (P =0.010) (43). Decreased MCPH1/BRIT1 protein and RNA expression levels, and/or deletions and inactivation due to methylation were reported in 96% (121/126) of breast cancer cases. Additionally, patients with MCPH1/BRIT1 inactivation by methylation showed a correlation with negative ER status. Moreover, the change in both MCPH1/BRIT1 and ATM together was significantly associated with an increased in tumour grade (44).

MCPH1/BRIT1 has also been identified as a novel hereditary breast cancer gene. In a study conducted to assess the expression of DNA damage proteins, 40% (50/125) of sporadic cases and 65.3% (47/72) of familial breast cancer cases demonstrated reduced MCPH1/BRIT1 expression which was also associated with higher tumour grade (45). The function of MCPH1/BRIT1 as a breast cancer susceptibility gene was supported by another study in which a recurrent heterozygous MCPH1/BRIT1 mutation c.904_916del was identified in 3.4% (5/145) familial and 1.4% (16/1150) sporadic breast cancer cases (46). Blood samples from MCPH1/BRIT1 mutation carriers also demonstrated high levels of chromosomal rearrangements suggesting MCPH1/BRIT1 haploinsufficiency causes increased genomic instability and cancer susceptibility (46). CRISPR gene editing was subsequently used to generate the cancer predisposing allele p.Arg304Valfs-Ter3 in the normal breast cell line MCF10A which removed the two C-terminal BRCT domains. This study showed that the MCPH1/BRIT1 mutation led to transcriptional changes causing a significant increase in migration and invasion as well as abnormal chromosomal condensation (47).

MCPH1/BRIT1 has been shown to control the protein stability of p53 in breast cancer cell lines by blocking murine double minute 2-mediated (MDM2) p53 ubiquitination (56). In MCPH1/BRIT1 depleted cells p53 expression levels were reduced which was found to promote cell proliferation and survival in normal (MCF10A) breast cells. Depletion of MCPH1/BRIT1 in normal MCF10A cells caused transformation into oncogenic cells. Furthermore, the overexpression of MCPH1/BRIT1 in the MCF7 breast cancer cell line suppressed proliferation both *in vivo* and *in vitro* (56). Interestingly, breast cancer patients who are carriers of the MCPH1/BRIT1 p.Arg304ValfsTer3 mutation also demonstrated p53 mutations in breast tumours (47).

### MCPH1/BRIT1 in colorectal cancer

Lack of heterozygosity (LOH) on chromosome 8p23.1, the MCPH1/BRIT1 locus has frequently been identified in advanced stage colorectal carcinoma patients, indicating the presence of a tumour suppressor gene. Approximately one-third of colorectal cancer cause microsatellite instability (MSI) because of the failure in mismatch repair (49). Frequently, tumour suppressor genes are targets for mutations at mononucleotide repeats within the genes in high MSI cancers (49). The MCPH1/BRIT1 gene contains a single nucleotide repeat (A9), which is a mutation target in cancers with MSI that inhibits the TSG activities contributing to tumorigenesis (49). Methylation of the MLH1 promoter is not an explanation for many colorectal cancers with MSI (50). Lynch syndrome is caused by germline mutations in other mismatch repair (MMR) genes (50). These Lynch-like syndrome patients, on the other hand, have somatic mutations in MMR genes (50). Xicola et al. discovered rare, likely pathogenic germline variants in repair genes that maintain genome integrity in Lynch-like syndrome, including a stop mutation in MCPH1/BRIT1 (50). Furthermore, interrogation of the Cancer Genome Atlas (TCGA; cBioPortal Cancer Genomics database http://cancergenome.nih.gov/) identified MCPH1/BRIT1 mutations in 7.4% of colorectal adenocarcinoma (**Figure 3**).

In cancer, centrosome amplification is common and associated with severe clinical features and poor patient outcomes (31). Interrogation of TCGA genomic and transcriptomic data identified 367 genes that encode proteins with centrosome localisation (31). Cellulo analysis of these candidates (633 colorectal cancer patients), demonstrated that the deletion of MCPH1/BRIT1 resulted in the most significant increase in centriole number (31). MCPH1/BRIT1 deletion, mutation and amplification have all been linked to colorectal cancer. MCPH1/BRIT1 deletion was discovered to be strongly linked to p53 abnormality (31). Patients with MCPH1/BRIT1 deletions have a poor outcome. The Kaplan–Meier curve shows that disease-free survival (DFS) is shorter in colorectal cancer patients with MCPH1/BRIT1 deletion than in patients with wild-type MCPH1/BRIT1 (P =0.025) (31). In addition, Zhang Du et al. (2020) discovered a link between Angpt2 (rs12674822) single nucleotide polymorphisms (SNPs) and colorectal cancer patients’ progression-free survival (PFS) (52). This SNP (rs12674822) is located in the Angpt2 and MCPH1/BRIT1 introns (52). In another study, they performed whole-exome sequencing on a cohort of 30 adults, 30 adolescent and young adult and 2 pediatric colon cancers (51). A statistically significant difference in mutational frequency between adolescent, young adult and adult samples was observed in 43 genes, including MCPH1/BRIT1 (51). Many of these mutations are damaging because they are nonsynonymous, missense, stop-gain, or frameshift mutations (51).

### MCPH1/BRIT1 in ovarian cancer

Previously we have reported reduced MCPH1/BRIT1 staining in 19% (7/36) of primary ovarian cancer cells cultured from ascites samples (53). This reduced expression was associated with increased tumour grade and poor prognosis. Building on this study we next examined MCPH1/BRIT1 expression in normal ovarian, endometrium and fallopian tube tissue. Strong nuclear MCPH1/BRIT1 staining was identified in ovarian epithelial cells by (90%) compared to endometrium and fallopian tube samples which are (70–80%) (27). Similarly, we identified reduced MCPH1/BRIT1 expression in 33% (84/252) of epithelial ovarian cancer tumour samples. Again, low MCPH1/BRIT1 expression was statistically associated with high grade tumours and advanced stage tumours (27). MCPH1/BRIT1 expression levels were not associated with different epithelial ovarian cancer subtypes. Consequently, the expression of MCPH1/BRIT1 could be a useful biomarker in epithelial ovarian cancer (27). This work complements studies which have identified decreased MCPH1/BRIT1 DNA copy number and mRNA levels in 40% (35/87) and 63% (19/30) of ovarian cancer respectively (54). In a further ovarian cancer study, the expression levels of the hTERT regulators were investigated as potential biomarkers to treat epithelial ovarian cancer. MCPH1/BRIT1 and the functional form of hTERT were associated negatively which in turn identified MCPH1/BRIT1 as a negative telomerase regulator in primary epithelial ovarian cancer samples (55).

### MCPH1/BRIT1 in lung cancer

Three lung cancer studies related to MCPH1/BRIT1 were identified, all of which were conducted in Chongqing, China (30, 56, 57). These studies have shown that the defect in the expression of MCPH1/BRIT1 may participate in the development of lung cancer. The initial study used immunohistochemistry to evaluate the expression of MCPH1/BRIT1 protein in two groups, 188 patients with lung cancer and 20 patients with normal lung tissues. In all normal lung samples positive MCPH1/BRIT1 staining was found. In the lung cancer samples significant lower numbers of MCPH1/BRIT1-positive cells were identified compared with normal tissues. The expression of MCPH1/BRIT1 varied among different histological subtypes, patients with lung adenocarcinoma expressed higher MCPH1/BRIT1 than patients with squamous cell lung carcinoma (56). A small follow up study confirmed significantly reduced MCPH1/BRIT1 mRNA expression in lung cancer samples compared to adjacent normal tissue. Functional studies were also performed in A549 lung cancer cells, which demonstrated over-expression of MCPH1/BRIT1 caused reduced proliferation due to cell-cycle arrest at S and G2/M and increased apoptosis (30).

A further study revealed that MCPH1/BRIT1 over-expression inhibits the migration and invasion capacities of A549 lung cancer cells by suppressing Snail and Slug proteins by blocking Mdm2-mediated p53 ubiquitination (57). Additionally, MCPH1/BRIT1 is an inclusion eligibility criterion in 2 clinical trials for squamous cell lung carcinoma, 3 clinical trials for non-small cell lung carcinoma, and 5 clinical trials for small cell lung carcinoma (72). All of these studies point to the possibility that MCPH1/BRIT1 functions as a tumour suppressor in the lungs.

### MCPH1/BRIT1 role in other different cancer types

We searched PubMed and found 16 studies identified the potential role of MCPH1/BRIT1 in different cancer types such as, lymphoma, chronic myeloid leukemia, cervical cancer, endometrial cancer, hepatocellular carcinoma, renal carcinoma, brain cancer, oral squamous cell carcinoma, melanoma and prostate cancer.

### MCPH1/BRIT1 in lymphoma and chronic myeloid leukemia

A study by Liang et al. used knockout mouse models, Mcph1 deficient and Mcph1/p53 deficient mice (61). They showed that Mcph1- and Mcph1-/p53- mice developed lymphomas earlier than in Mcph1^+^/p53¯ mice. By using metaphase spread assay and spectral karyotyping analysis they concluded that Mcph1 depletion regulates genomic instability and induces carcinogenesis. Giallongo et al. investigated genomic instability and the ability of chronic myeloid leukemia cells to arrest mitotic division following exposure to the genotoxic drug hydroxyurea (62). In agreement with the key role of MCPH1/BRIT1 in the regulation of cell cycle progression at the G2/M checkpoint, they found that chronic myeloid leukemia cells have a low level of MCPH1/BRIT1 and a defective G2/M arrest, confirming the genomic instability of these cells. In addition, A study showed that downregulation of MCPH1/BRIT1 accompanied by loss of its promoter methylation resulted in upregulation of ANGP2 which is a crucial factor in tumour angiopoiesis in chronic lymphocytic leukemia (63).

### MCPH1/BRIT1 in cervical endometrial cancer

An investigation of MCPH1/BRIT1 in cervical cancer demonstrated decreased expression of MCPH1/BRIT1 in 61.3% (19/31) of cases at the mRNA level and 69.8% (44/63) at the protein level in cervical tumour tissues compared to non-tumour tissues. The downregulation of MCPH1/BRIT1 correlated with increased tumour grade (29). They also demonstrated that overexpression of MCPH1/BRIT1 induced S phase arrest and mitochondrial apoptosis (29). Moreover, a study by Bilbao et al. investigated whether endometrial cancer is caused by DSB repair with MSI (64). Mononucleotide microsatellite tracts of 14 genes of the DSB repair system including MCPH1/BRIT1 were analyzed in a series of 41 endometrial cancers with MSI. Mutations in MCPH1/BRIT1 were detected in 12% (5/41) of endometrial cancer indicating that MCPH1/BRIT1 could be a target gene of MSI (64).

### MCPH1/BRIT1 in gastric carcinoma

Similarly, investigation of MCPH1/BRIT1 frameshift mutations in gastric cancer found that 11% (4/34) of cases with high MSI contained MCPH1/BRIT1 frameshift mutations compared to (0/45) with low MSI (49). Another study characterising long non-coding RNAs (lncRNA) in gastric cancer identified over-expression of 6 key lncRNA including MCPH1/BRIT1 antisense RNA 1 (CTD-2541M15) which were associated with invasion and metastasis (58).

### MCPH1/BRIT1 in hepatocellular carcinoma

Scientists identified that MCPH1/BRIT1 deficiency is a potential factor in hepatocellular carcinoma (HCC) development. HCC samples were analyzed for MCPH1/BRIT1 alterations at DNA, RNA, and protein levels (65). Deletion and/or downregulation of MCPH1/BRIT1 was found in ~30% of HCC samples. Notably, the lack in survival ratio and increase in the recurrence of HCC were caused by MCPH1/BRCA1 deficiency (65). The K659fsX10 mutation in MCPH1/BRIT1 represented in HCC blocked DNA repair function. Moreover, MCPH1/BRIT1 depleted-HCC cells were HR defective and more sensitive to PARPi olaparib alone or plus PI3K inhibitor BEZ235. Ectopic MCPH1/BRIT1 significantly decreased the cytotoxicity of olaparib alone or with BEZ235. In addition, in MCPH1/BRIT1 depleted cells, BEZ235 induced poly (ADP-ribose) release, DSB ratio and single-strand breaks (65). MCPH1/BRIT1 deficiency is therefore a regulator for HCC growth and using olaparib and/or BEZ235 as a treatment is highly sensitive in MCPH1/BRIT1 deficient HCC. PI3K blocking is considered a major cause for DNA damage and MCPH1/BRIT1 depleted-cells become more dependent on PARP activity, thus, the synthetic lethal ability of PARP and PI3K inhibitors is enhanced by the deficiency of MCPH1/BRIT1 in HCC (65).

### MCPH1/BRIT1 in renal cancer

A study evaluated the expression of MCPH1/BRIT1 in 188 renal cancer and 20 normal renal tissues by immunohistochemistry (66). They found that all normal renal samples displayed positive MCPH1/BRIT1 staining while renal carcinoma tissues showed lower levels of MCPH1/BRIT1 staining compared to normal tissues. Functional analysis in renal cancer cell lines demonstrated that over-expression of MCPH1/BRIT1 reduced proliferation, migration and invasion while increasing apoptosis supporting MCPH1/BRIT1’s role as a tumour suppressor gene in renal cancer. The down regulation of MCPH1/BRIT1 expression was also shown to be correlated with miRNA-27a and linked to miRNA-27 seed regions identified in MCPH1/BRIT1 3’UTR (66).

### MCPH1/BRIT1 in brain cancer

MCPH1/BRIT1 promoter methylation was identified in 96.6% (28/30) of brain tumour samples (67). In a follow up study MCPH1/BRIT1 promoter methylation was determined in 14 paired circulating cell-free DNA (cfDNA) from serum samples and genomic DNA from tumour tissue also 18 isolated serum samples with different grades of brain tumours. MCPH1/BRIT1 promoter methylation was identified in 78% of tissue samples and 54% cfDNA samples with agreement between samples in 57%. MCPH1/BRIT1 promoter methylation was also significantly associated with high tumour grade suggesting it could be an epimarker in the detection of brain tumours (67). In contrast, it was found that the expression of MCPH1/BRIT1 did not alter during glioma development (59).

### MCPH1/BRIT1 in oral squamous cell carcinoma

In other cancer types such as oral squamous cell carcinoma (OSCC), LOH was performed in a panel of 81 matched normal oral tissues and OSCC samples and observed that 19.72% (14/71) informative samples showed LOH at 8p23 (60). MCPH1/BRIT1 was downregulated at the transcript and protein levels in 51.22% (21/41) and 76% (19/25) of OSCC samples respectively. MCPH1/BRIT1 over-expression in oral squamous KB cells reduced proliferation, cell invasion and anchorage-independent growth and tumour growth in nude mice (60). This study also demonstrated that miR-27a targets MCPH1/BRIT1 and negatively regulates its levels (60). In a recent study, the 5-year survival of patients with early-stage (I/II) OSCC was predicted by combining clinicopathologic features with a gene methylation signature. The most significant methylation difference between patients who lived for over five years and those who died was found in MCPH1/BRIT1. Therefore, indicating MCPH1/BRIT1 gene methylation predicts poor prognosis in early-stage (I/II) OSCC (68).

### MCPH1/BRIT1 in melanoma

The involvement of MCPH1/BRIT1 dysfunction was suggested in the development and progression of cutaneous melanoma. Immunohistochemistry was performed identifying abnormal MCPH1/BRIT1 expression in 10% (1/10) of melanocytic nevi and in 86.3% (44/51) of primary cutaneous melanomas (69).

### MCPH1/BRIT1 in prostate cancer

A number of pan-cancer studies have reported an association between MCPH1/BRIT1 and prostate cancer. The MCPH1/BRIT1 human chromosomal locus 8p23.1 was previously described as deleted in prostate cancer and associated with a poor prognosis. A small prostate study showed a reduction in MCPH1/BRIT1 protein levels in cancer samples compared to normal tissues (54). Bioinformatics analysis performed on TCGA datasets to identify centrosome genes associated with centrosome amplification and cancer development found that prostate adenocarcinoma cases with MCPH1/BRIT1 gene deletion demonstrated a worse OS and DFS (31). Importantly MCPH1/BRIT1 gene dysregulation has been identified in 12-16% of prostate cancer cases suggesting MCPH1/BRIT1 warrants further study in this cancer type (31).

### Despite significant advances in cancer treatment, novel therapeutic strategies are required

Personalized medicine became a reality after the human genome project was finished in 2001. Understanding the genetic code would pave the way for the development of anti-cancer medications that directly target dysregulated pathways. As an illustration, personalized medicine has dramatically reduced mortality for several cancer types. Nowadays, clinical practice utilizes commercially available genetic testing to direct treatment decisions (73, 74). In general, developments in cancer treatment have resulted in better patient outcomes; given the clinical relevance of MCPH1/BRIT1 expression we anticipate that MCPH1/BRIT1 defective patients would benefit from targeted therapies.

Immunotherapy has become an important therapeutic option for many malignancies particularly advanced stage cancers. A number of features have been identified which predict if the use of immune checkpoint inhibitors (ICIs) will be successful. For example, tumours with homologous recombination defects, with high expression of PD-1 and PD-L1, represent better susceptibility to ICIs (75). Also using PARPi synergy with the ICIs in tumours with homologous recombination damage reported increases in the mutational load in tumour cells and increased expression of PD-L1 by ATM-ATR-Checkpoint kinase 1 pathway (76, 77). In a small renal cell carcinoma study, loss of function mutation in one component of the SWI/SNF (PBRM1) complex, demonstrated a better response to anti-PD-L1 therapy (78). Also, high-throughput CRISPR screening identified an association between SWI/SNF complex genes and better response to ICIs (79). These previous studies suggest a rationale to use ICIs with MCPH1/BRIT1-deficient cancers.

Another promising approach to investigate would be MCPH1/BRIT1 synthetic lethality. MCPH1/BRIT1 deficiency facilitates the synthetic lethality of PARP and PI3K inhibitors in HCC (65). This provides a new biological foundation for dramatically expanding PARPi’s therapeutic potential in various types of cancer with depletion in MCPH1/BRIT1. It is critical to discover new compounds and genes that cause synthetic lethality in MCPH1-deficient cells. The identification of these targets and molecules, as well as research of the cellular pathways in which they are engaged, may aid in cancer treatment discovery.

## Conclusions

In conclusion, the common findings of the role of MCPH1/BRIT1 in tumorigenesis in various types of cancer, is that the loss of MCPH1/BRIT1 gene plays a key role in promoting genome instability and mutations, which is one of cancer’s hallmarks. Consequently, future studies are warrants to examine MCPH1/BRIT1 as a tumour suppressor.

## Author contributions

MA designed the review, directed and took the lead in writing the manuscript. All authors contributed to the article and approved the submitted version. RA devised the review and proof outline. SB provided critical feedback and helped shape the manuscript.
